# Lipofibromatosis arising in a right labiocrural fold in a 14-month-old female: a case report study with a 3-year follow-up

**DOI:** 10.1093/jscr/rjad728

**Published:** 2024-01-10

**Authors:** Zlatan Zvizdic, Emir Milisic, Nurija Bilalovic, Nermina Ibisevic, Melika Bukvic, Semir Vranic

**Affiliations:** Department of Pediatric Surgery, Clinical Center University of Sarajevo, Sarajevo 71000, Bosnia and Herzegovina; Department of Pediatric Surgery, Clinical Center University of Sarajevo, Sarajevo 71000, Bosnia and Herzegovina; Department of Pathology, Clinical Center University of Sarajevo, Sarajevo 71000, Bosnia and Herzegovina; Department of Pathology, Clinical Center University of Sarajevo, Sarajevo 71000, Bosnia and Herzegovina; Department of Radiology, Clinical Center University of Sarajevo, Sarajevo 71000, Bosnia and Herzegovina; College of Medicine, Q.U. Health, Qatar University, Doha 2713, Qatar

**Keywords:** lipofibromatosis, diagnosis, surgery, histopathology, children

## Abstract

Lipofibromatosis (LF) is a rare benign fibrofatty tumor of infancy and childhood with a predilection for distal extremities, poor margination, and a high local recurrence rate. We report a toddler who presented with an LF involving her right labiocrural fold. Imaging showed a soft tissue mass extending through the right labiocrural fold with possible infiltration into the underlying muscles. The mass was excised entirely, preserving adjacent structures. The histopathologic report revealed the mass to be LF. A 3-year follow-up revealed no disease recurrence. No other cases of LF in this localization have been presented in the literature. Despite its rarity, LF should be considered in diagnosing soft tissue neoplasms in children. Accurate diagnosis and proper surgical management with complete resection are essential to reduce the postoperative recurrence risk.

## Introduction

Lipofibromatosis (LF), or nondesmoid-type infantile fibromatosis, is a rare soft tissue pediatric tumor first described as a separate entity by Fetsch *et al*. in 2000 [1]. LF was first recognized as a distinctive fibrofatty tumor of childhood in the World Health Organisation (WHO) classification of soft tissue tumors in 2002 [2]. The etiology and risk factors for LF have not been established. The tumor is exclusive to children and is sometimes congenital. The most common location of LF is the subcutaneous tissues of the upper and lower distal extremities and, less frequently, of the trunk, the head, and the neck region [3]. LF is a slow-growing, painless mass in infants and children, from newborns to teenagers, with a 2.7:1 male-to-female distribution [1]. LF is notorious for its histologically benign appearance and high local recurrence incidence (33%–72%) [1, 4]. Due to its radiologic and clinical nonspecificity, a definitive LF diagnosis is mainly based on histopathologic findings. Histologically, LF is composed of abundant, usually mature, adipose tissue traversed by bundles of spindle-shaped fibroblast-like cells, often concentrated in septal and perimysial locations [2]. Clinically, differential diagnoses include hemosiderotic fibrolipomatous lesion, neural fibrolipoma, juvenile fibromatosis, fibrous hamartoma of infancy (primitive oval cell component with myxoid stroma), lipoblastoma/lipoblastomatosis, and calcifying aponeurotic fibroma [5]. Since no effective medical therapy has been found for this type of lesion and no spontaneous regression has been reported, complete surgical resection with the preservation of adjacent neurovascular structures is the mainstay of treatment in children with initial and locally recurrent LF [1].

Herein, we report a case of a 14-month-old baby who presented with a 7-month history of a slowly enlarging, painless mass of the right labiocrural fold together with the radiologic and histopathologic findings.

## Case report

**Figure 1:**
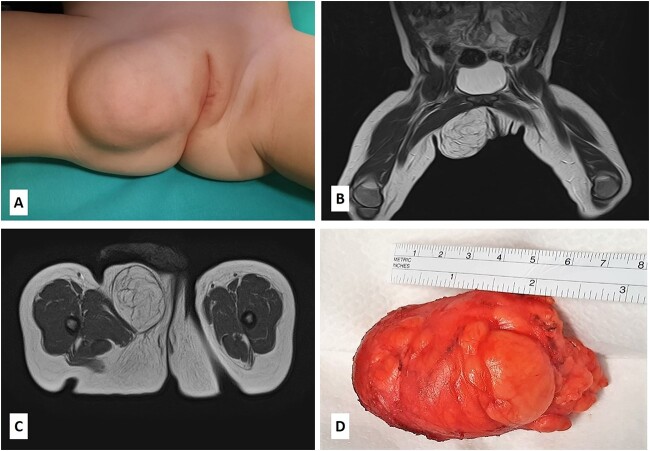
(A) The patient presented with a nontender, mobile, round mass of the right labiocrural fold; (B–C) MRI scan revealed a not well-demarcated mass measuring 6.6 × 4.8 × 4.4 cm; the mass mainly comprised fatty signal intensity; (D) the gross appearance of the specimen showing a lobulated, solid fatty mass with a thin, fibrous capsule.

**Figure 2:**
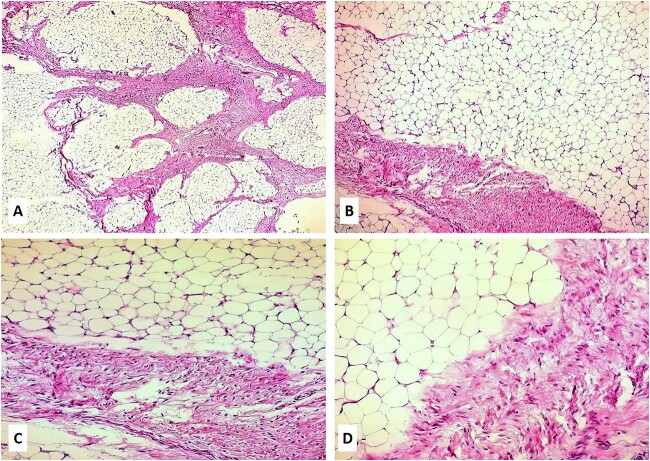
(A–D) Hematoxylin and eosin slides of the biopsy revealed a neoplasm composed of lobules of mature adipose tissue admixed with fibroblastic foci consisting of bland fibroblasts involving adipose septa with preserved lobular architecture; magnifications: 5× (A), 10× (B), 20× (C), and 40× (D).

A 14-month-old girl with a 7-month history of a slowly enlarging, painless right groin/right labial mass was referred for assessment to our institution. There was no history of trauma or infection. Physical examination revealed a 6 × 4-cm nontender, mobile, round mass of the right labiocrural fold (Fig. 1A). No overlying skin changes were noted. All laboratory tests were normal. The plain radiograph was unremarkable. Soft tissue ultrasound exhibited a well-defined, slightly hyperechoic mass of the right labiocrural fold. Doppler evaluation stated a minimal color Doppler flow. Magnetic resonance imaging (MRI) revealed a not well-demarcated mass measuring 6.6 × 4.8 × 4.4 cm (Fig. 1B and C). The mass mainly comprised fatty signal intensity (Fig. 1B and C). With a presumed diagnosis of fibrous hamartoma or lipoblastoma, the patient underwent surgical exploration of the mass. An incision is made over the right labium majus superiorly and extending inferiorly to the level of the right labiocrural fold. A lobulated, solid fatty mass with a thin, fibrous capsule and without focal cystic or hemorrhagic change was densely adherent to muscle fibers of the adductor muscles of the medial thigh. The mass was completely excised, with the resulting specimen of 7 × 5 × 4.5 cm (Fig. 1D). The histopathologic examination revealed a tumor composed of lobules of mature adipose tissue admixed with fibroblastic foci consisting of bland fibroblasts involving adipose septa with a preserved lobular architecture. No prominent atypia or mitotic figures were observed (Fig. 2A–D). Immunohistochemically, the spindle cells were positive for CD34 and CD99, with a weak and focal positivity for EMA and bcl-2. The fibroblasts were negative for S-100 and ß-catenin (only cytoplasmic, no nuclear expression). These morphologic findings were consistent with LF.

The patient’s postoperative course was uneventful. A 3-year follow-up (clinical and radiologic) revealed no evidence of recurrence.

## Discussion

The rarity and diversity of benign fibrous/fibrolipomatous tumors in infancy and childhood present a diagnostic and therapeutic challenge. LF was first described as a separate entity in 2000 [1] and was included in the WHO classification under fibroblastic/myofibroblastic tumors in 2002 [2]. It was finally reclassified under the category of intermediate-locally aggressive fibroblastic/myofibroblastic tumors in 2013 [3, 6].

LF is a slow-growing, painless tumor of infancy and early childhood which usually affects the subcutaneously or the deep soft tissues of the distal extremities but may involve the thigh, trunk, or head [3]. Although they generally do not impair function, they can infiltrate adjacent neurovascular bundles and muscles [7]. The size of LF varies from 1 to 7 cm [7]. The clinical features of the present case were consistent with those previously reported. It is essential to recognize the characteristics of this entity for proper diagnosis and clinical management.

Radiologic imaging techniques that can be used for preoperative assessment include Doppler ultrasound, computed tomography, and MRI. Preoperative imaging helps assess the extent of the tumor. However, an accurate preoperative diagnosis of LF is rarely made due to the radiologic overlap of LF with other fibrofatty lesions [8]. MRI is considered as the imaging modality of choice in diagnosing LF. It typically shows LF as a lipomatous lesion with varying amounts of adipose and solid components without a visible capsule on its periphery [8]. The appearance of LF as a type of fibrofatty tumor on MRI is characterized by a high signal on T1- and T2-weighted sequences with fat saturation [9]. For postoperative surveillance, MRI is the currently recommended modality for follow-up, particularly in cases of incomplete resection or prior recurrence.

The differential diagnosis other than LF includes hemosiderotic fibrolipomatous lesion, neural fibrolipoma, juvenile fibromatosis, fibrous hamartoma of infancy, lipoblastoma/lipoblastomatosis, and calcifying aponeurotic fibroma [5]. In general, the definitive diagnosis of LF is usually made based on the analysis of the surgical specimen. Macroscopically, the excised tumor is most commonly described as yellow or brown/white with a firm, rubbery, or gritty texture, and with fat, that is often noted grossly [1, 2]. Microscopically, LF is composed predominantly of mature adipose tissue with a spindled fibrous tissue element traversing the adipose tissue in the form of septa [10]. By contrast, juvenile fibromatosis typically exhibits a solid sheet-like fibrous growth pattern with minimal or no fat tissue. It also has a characteristic *CTNNB1* mutation in ~90% of cases, followed by the nuclear translocation of ß-catenin expression, which was not observed in our case. Another important differential diagnosis is infantile fibrous hamartoma, which is composed of primitive oval cells with myxoid stroma; the features were not seen in our case.

Complete surgical resection with preservation of neurovascular structures is the treatment of choice because of the predilection for recurrence in incompletely excised lesions [1, 2]. However, the treatment of LF should be individualized based on the clinical presentation because there are cases in which long-term follow-up has shown no progress of the tumor despite incomplete excision [11]. Although no metastatic potential has been described, LF is burdened by a high local recurrence rate despite its benign nature. The regrowth and persistent disease rate is between 33% and 72%, particularly in cases of incomplete resection or prior recurrence [1, 4]. Our literature survey revealed no similar case of this anatomic localization in the current literature.

Although very rare, LF should be considered in diagnosing soft tissue neoplasms in children. Despite the growing number of reported cases, there is still insufficient clinical experience about this pathological condition of childhood and a lack of awareness of its existence. With inconclusive preoperative imaging findings and the absence of an accurate preoperative diagnosis of LF in most cases, complete surgical resection remains the only chance for curative therapy.

## Data Availability

Data available from the corresponding authors on a reasonable request.
